# Mechanisms underlying the role of ankyrin-B in cardiac and neurological health and disease

**DOI:** 10.3389/fcvm.2022.964675

**Published:** 2022-08-04

**Authors:** Nicole S. York, Juan C. Sanchez-Arias, Alexa C. H. McAdam, Joel E. Rivera, Laura T. Arbour, Leigh Anne Swayne

**Affiliations:** ^1^Division of Medical Sciences, University of Victoria, Victoria, BC, Canada; ^2^Department of Medical Genetics, University of British Columbia, Victoria, BC, Canada; ^3^Department of Cellular and Physiological Sciences and Djavad Mowafaghian Centre for Brain Health, University of British Columbia, Vancouver, BC, Canada

**Keywords:** scaffolding protein, cellular morphology, calcium homeostasis, excitation-contraction coupling, arrhythmia, sudden cardiac death, seizure, autism spectrum disorders

## Abstract

The *ANK2* gene encodes for ankyrin-B (ANKB), one of 3 members of the ankyrin family of proteins, whose name is derived from the Greek word for anchor. ANKB was originally identified in the brain (B denotes “brain”) but has become most widely known for its role in cardiomyocytes as a scaffolding protein for ion channels and transporters, as well as an interacting protein for structural and signaling proteins. Certain loss-of-function *ANK2* variants are associated with a primarily cardiac-presenting autosomal-dominant condition with incomplete penetrance and variable expressivity characterized by a predisposition to supraventricular and ventricular arrhythmias, arrhythmogenic cardiomyopathy, congenital and adult-onset structural heart disease, and sudden death. Another independent group of *ANK2* variants are associated with increased risk for distinct neurological phenotypes, including epilepsy and autism spectrum disorders. The mechanisms underlying ANKB's roles in cells in health and disease are not fully understood; however, several clues from a range of molecular and cell biological studies have emerged. Notably, ANKB exhibits several isoforms that have different cell-type–, tissue–, and developmental stage– expression profiles. Given the conservation within ankyrins across evolution, model organism studies have enabled the discovery of several ankyrin roles that could shed important light on ANKB protein-protein interactions in heart and brain cells related to the regulation of cellular polarity, organization, calcium homeostasis, and glucose and fat metabolism. Along with this accumulation of evidence suggesting a diversity of important ANKB cellular functions, there is an on-going debate on the role of ANKB in disease. We currently have limited understanding of how these cellular functions link to disease risk. To this end, this review will examine evidence for the cellular roles of ANKB and the potential contribution of ANKB functional variants to disease risk and presentation. This contribution will highlight the impact of ANKB dysfunction on cardiac and neuronal cells and the significance of understanding the role of ANKB variants in disease.

## Introduction

Loss-of-function variants in the *ANK2* gene are associated with a wide range of electrical and structural heart disease. Reported cardiac phenotypes include arrhythmia, corrected QT interval prolongation (sometimes referred to as long QT type 4), and sudden cardiac death. A prolonged QT interval on an electrocardiogram corrected for heart rate (QTc) is a predictor of ventricular arrythmias and sudden cardiac death (1, 2). At least 15 congenital long QT syndromes (LQTS) have been described, associated with genes encoding for ion channels, ion channel modulatory subunits, signaling proteins, and cytoskeleton-associated proteins (3). One of the first identified *ANK2* variants, p.E1458G, was associated with prolonged QTc, and this QTc prolongation has since been associated with other *ANK2* variants (4–7). Notably, a prolonged QTc is not observed in all patients harboring cardiac phenotype- associated *ANK2* variants. In fact, there is minimal evidence of a prolonged QTc in individuals under the age of 25 (7). Additional reported cardiac manifestations include bradycardia, sinus arrhythmia, idiopathic ventricular fibrillation, and catecholaminergic polymorphic ventricular tachycardia (5, 8). Separately, *ANK2* is also emerging as a gene of interest in neurological disorders. *ANK2* has been identified as a key risk gene for autism spectrum disorders (ASD) (9, 10) and as a candidate gene for epilepsy (11).

The protein produced from the *ANK2* gene, ankyrin-B (ANKB), is a large scaffolding protein that has become known as a key regulator of cardiac physiology (4, 12). There are three mammalian ankyrin protein family members, including ANKB, ankyrin-R (ANKR, *ANK1* gene), and ankyrin-G (ANKG, *ANK3* gene). ANKR is primarily expressed in erythrocytes (13) while ANKB and ANKG are co-expressed in a variety of cell types and tissues (14–16). Ankyrins, including ANKB, are composed of four domains: a membrane binding domain comprised of 24 *ANK* repeats that interacts with membrane proteins such as ion channels and transporters, a spectrin binding domain responsible for interacting with βII spectrin, a death domain of which the function has not yet been identified but in other proteins is key for signal transduction cascades resulting in apoptosis and inflammation (17), and a C-terminal domain. The death domain and C-terminal domain comprise the regulatory domain which is named due to its ability to directly bind the membrane binding domain and play a role in inhibition (15). As this review is focused on ANKB, the following information is specific to ANKB, except where information about other ankyrin family members provides key insight.

*ANK2* has critical roles for cardiac and neuronal physiology as indicated by loss-of-function variants and studies using model organisms. ANKB's structure and different isoforms allow for a diverse array of protein-protein interactions within a variety of different cell types. As such, dysfunction in ANKB can lead to a wide range of cellular impacts. There are different groups of variants associated with different phenotypes; one group of *ANK2* variants is primarily associated with a broad cardiac phenotype, another is associated with neurological diseases including ASD and epilepsy, and others are linked to metabolic perturbations. The *ANK2* variant-associated clinical phenotypes inform investigation of ANKB cellular roles, including key potential protein-protein interactions and cellular processes that could, in turn, help to develop new therapeutic strategies. To this end, we first highlight certain *ANK2* variants associated with disease and then discuss the potential underlying mechanisms garnered from cell biological studies using a variety of model organisms. These studies have revealed key cellular roles for ANKB in the localization and spatial organization of ion channels and transporters, signaling molecules, and structural proteins involved in variety of cellular processes, including development of cellular morphology, calcium homeostasis, and glucose and fat metabolism. By linking ANKB's emergent cellular roles with phenotypes associated with *ANK2* variants, a picture of ANKB's many contributions to cardiac, neurological, and metabolic health and disease begins to emerge. Making these links is key to translating this knowledge into the clinical setting and helps understand disease risk and presentation.

## Tissue- and cell-type-specific expression of ANKB isoforms across development

There are several ANKB isoforms which exhibit cell-type–, tissue–, and developmental stage–specific expression patterns. While the 220 kDa ANKB isoform is the primary isoform in both the heart and brain [as well as other cells and tissues, such as skeletal muscle, thymus, pancreas, and adipose tissue (18, 19) certain isoforms exhibit tissue-specific expression. The initial discovery of *ANK2* (and its product ANKB) resulted from a series of studies characterizing ankyrin cDNA enriched in non-erythroid cells (20, 21). After the identification of a 440 kDa isoform, consisting of a large insertion (exon 40) between the regions encoding for the spectrin binding domain and death domain (20, 22), transcript and protein level characterization showed that 440 kDa ANKB was detectable at birth, with expression levels peaking at postnatal day 10 and decreasing progressively in the adult rat brain (down to 30% of peak levels) (22). Meanwhile, the 220 kDa ANKB transcript and protein levels were found to increase progressively through development into adulthood (20, 23). In addition to the 220 kDa isoform, additional ANKB isoforms have been detected in the heart: a 188 kDa isoform that, similarly to 220 kDa ANKB isoform, when knocked down results in altered expression and localization of the sodium calcium exchanger, a 212 kDa isoform which is localized to striated muscle and the cardiac M-line (24), and a 160 kDa isoform that is highly expressed in mouse hearts along with the 220 kDa isoform (25).

Newer transcriptomics studies and databases of the developing human heart show that *ANK2* is differentially expressed in human embryonic ventricular and atrial cardiomyocytes, with high transcript levels also detected in fibroblast-like cells associated with vascular development and cardiac neural crest cells (https://spatialtranscriptomics3d.shinyapps.io/Developmental_heart_explorer/) (26). *ANK2* transcript levels peak during early mid-fetal human development (and mouse *Ank2* transcript levels peak during late embryonic development) to eventually plateau during later developmental stages (https://hbatlas.org/mouseNCXtranscriptome/, https://hbatlas.org/pages/hbtd) (26–29). In the 1-week-old mouse brain, *Ank2* transcript levels are enriched in astrocytes, neurons, and oligodendrocyte progenitors (https://www.brainrnaseq.org/) (30). At the single RNA-seq level, *ANK2* transcript levels are slightly enriched in inhibitory and excitatory neuron populations [Allen Cell Type Database – “M1 - 10X GENOMICS (2020)”; https://portal.brain-map.org/atlases-and-data/rnaseq] (31). Consideration of this ANKB enrichment in select cell types, tissues, and developmental stages could help provide important clues to the clinical impacts of *ANK2* variants. In the next section, we will highlight several *ANK2* variants and associated phenotypes that provide important areas of focus for investigation of ANKB's cellular roles.

## *ANK2* variants and risk for disease

Consistent with enriched ANKB expression in heart and brain, a number of *ANK2* variants have been associated with a range of cardiac phenotypes while others are associated with neurological or metabolic phenotypes.

### ANK2 variants associated with (primarily) cardiac phenotypes

**Figure 1:**
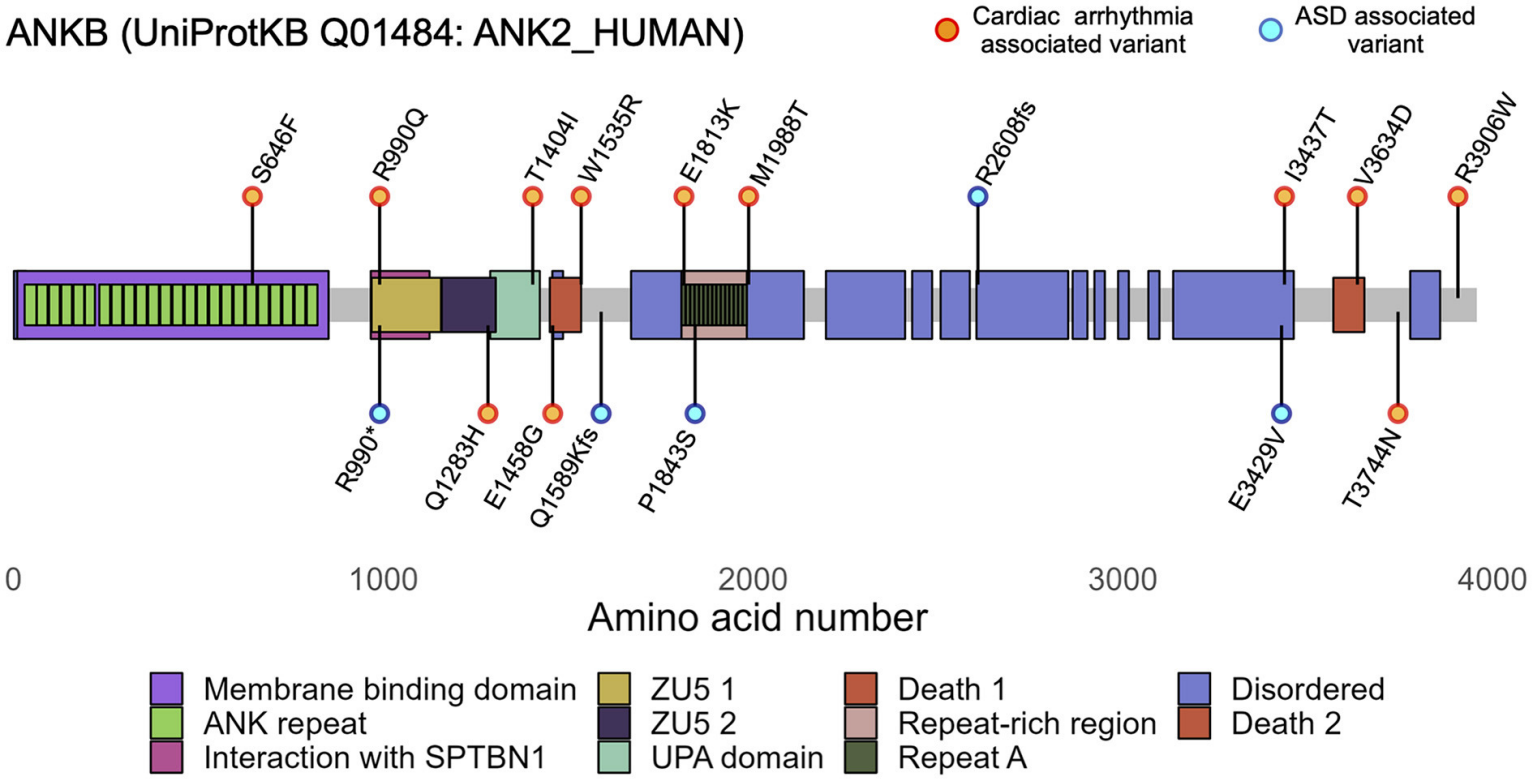
*ANK2* variants are associated to cardiac arrhythmias and autism spectrum disorders (ASD). Diagram of canonical human ANKB (UniProtKB Q01484-4, 3,957 aa) showing ANKB regions and domains. Canonically, ANKB can be divided into 4 main domains: an initial membrane binding domain, a spectrin binding domain, a death domain, and a C-terminus domain. The ANKB membrane binding domain contains 24 consecutive ankyrin (ANK) repeats and associates with ion channels and transporters; the spectrin binding domain contains highly conserved ZU5-ZU5-UPA domains and associates with *β*II-spectrin and the B56α subunit of PP2A; and the regulatory domain comprised of the death domain and C-terminus domain comprised is unstructured and highly variable between species. Orange and cyan dots represent the main cardiac and ASD variants discussed in the main text. Functional variants have been identified in each of ANKB's domains.

Certain *ANK2* loss-of-function variants are associated with a broad spectrum of cardiac phenotypes including arrhythmia, conduction abnormalities, and cardiomyopathy (Table 1) (4, 8, 32). Amino acid changes produced by these variants are present in all four domains of ANKB and are associated with autosomal dominant inheritance, reduced penetrance, and variable expressivity (Figure 1) (32). Initially described as LQTS type 4, QTc prolongation is commonly linked with cardiac-phenotype associated *ANK2* variants, although the role in QT prolongation has been since debated (33) (Table 1). ANKB p.E1458G (previously p.E1425G), the result of an amino acid substitution in the spectrin binding domain, was among the first *ANK2* variants identified. It was found in a French kindred with LQTS associated with atrial fibrillation and sinus node dysfunction (4). There was a family history of sudden death, including an 18 and 12 y.o. The variant demonstrated incomplete penetrance in one out of 23 carriers. Age related effects were also observed, affected children had sinus node abnormalities (diagnosed *in utero*) whereas atrial fibrillation was present only in adults (4). Of note, the p.E1458G variant has also been identified in a healthy Danish exome cohort without evidence of QTc prolongation and has a frequency of 0.11% (41/35360) in the Latino population according to the Genome Aggregation Database (gnomAD) (34, 35). Similarly, while two *ANK2* variants p.E1458G and p.V3634D (initially reported as p.V1516D) were over-represented in a private cohort from an inherited heart rhythm clinic, most patients carrying *ANK2* variants that were referred to this clinic showed no symptoms or had electrocardiographic findings of unknown significance; however, their genetic ancestry composition and clinical and epidemiological information is not publicly available (36). Another variant associated with prolonged QTc and ventricular tachyarrhythmias is the *ANK2* p.L1622I variant, found with higher frequency in individuals of African ancestry (minor allele frequency: 0.03, 850/24964, gnomAD) (5, 37).

**Table 1:** Spectrum of cardiac features associated with *ANK2* variants in humans.

		**E1458G^a^** ***N* = 25**	**R990Q** ***N* = 2**	**V3634D^b^** ***N* = 4**	**S646F** ***N* = 15**	**E1813K^c^** ***N* = 3**	**Q1283H** ***N* = 1**	**T1404I** ***N* = 1**	**M1988T** ***N* = 5**	**T3744N^d^** ***N* = 10**	**R3906W^e^** ***N* = 2**	**I3437T** ***N* = 1**	**W1535R** ***N* = 6**	**46,XX,t (4;9) (q25;q31.1)** ***N* = 5**
Variant type		Missense	Missense	Missense	Missense	Missense	Missense	Missense	Missense	Missense	Missense	Missense	Missense	LOF
Location		SBD	SBD	DD	MBD	CTD	SBD	SBD	CTD	CTD	CTD	Disordered	DD	n/a
Arrhythmia	LQTS	X	X		X					X	X		X	
	Drug-induced LQTS			X		X		X					X	
	CPVT			X								X		
	Atrial Fibrillation	X				X		X						
	Cardiac arrest		X	X								X		
	SCD	X			X				X	X				
	Bradycardia			X	X	X		X					X	X
	VT			X			X		X		X			
	Other arrhythmia		Recurrent VF	Type 1 brugada pattern	SVT				Late potential on SAECG		Torsades de pointes		Torsades de pointes, VF, BrS	
Conduction abnormalities	WPW				X									
	SND	X												X
	Other					Heart block								
Symptoms	Syncope	X	X	X	X	X	X		X	X	X		X	X
	Palpitations						X							
Structural	HCM	X	X									X		
	DCM				X									
	ARVC	X							X					
	Other structural													LV dysfunction, cardiomegaly
	Congenital Heart Defect				X									X
Other	Seizures				X								X	
GnomAD		0.0005346	0.00001971	0.002051	Not observed	0.002286	Not observed	Not observed	Not observed	0.0007363	0.0009337	Not observed	0.00001314	N/a
Clinvar Classification (# labs)		LP (3) VUS (4) LB (2)	VUS (3)	VUS (2) LB (5) B (1)	P (1)	VUS (1) LB (5) B (5)			VUS (1)	VUS (4) LB (7)	VUS (1) LB (6) B (1)		LP (1) VUS (1)	
ClinVar ID		VCV0000 18056.18	VCV000 190560.9	VCV0000 67599.12	VCV000 190552.2	VCV0000 18060.14			VCV00 1341732.1	VCV0000 18057.26	VCV0000 18059.23		VCV0000 67596.4	
Reference		(4, 44)	(6, 43)	(8)	(7)	(130)	(95)	(8)	(44)	(5)	(5)	(40)	(46)	(39)

SBD, Spectrin Binding Domain; DD, Death Domain; MBD, Membrane Binding Domain; CTD, C-Terminal Domain; LQTS, Long QT Syndrome; CPVT, Catecholaminergic Polymorphic Ventricular Tachycardia; SCD, Sudden Cardiac Death; VT, Ventricular Tachycardia; VF, Ventricular Fibrillation; SVT, Supraventricular Tachycardia; SAECG, Signal Averaged ECG; BrS, Brugada Syndrome; WPW, Wolff Parkinson White; SND, Sinus Node Dysfunction; HCM, Hypertrophic Cardiomyopathy; DCM, Dilated Cardiomyopathy; ARVC, Arrhythmogenic Right Ventricular Cardiomyopathy; P, Pathogenic; LP, Likely pathogenic; VUS, Variant of Uncertain Significance; LB, Likely benign; B, Benign.

a Previously E1425G;

b Previously V1516D;

c Proband also carried variant in KCNH2-H562R, only the phenotype in those carrying the ANK2 E1813K variant alone described in table. See text for description of the combined KCNH2 and ANK2 phenotype;

d Previously T1626N;

e Previously R1788W.

ANKB p.S646F, the first identified variant located in the membrane binding domain, also came to attention due to LQTS. This variant was found in two large multigenerational Gitxsan families identified because of LQTS in the context of a known high community prevalence of *KCNQ1*-mediated LQTS. The probands in each family did not carry the known *KCNQ1* variant (38), but instead, carried the p.S646F variant (7). As with the p.E1458G variant, QTc prolongation was not the only associated feature. The variant was identified in one individual who died suddenly due to dilated cardiomyopathy, another carrier had a history of Wolf-Parkinson-White (WPW) syndrome, and this individual's daughter was born with a congenital heart defect (total anomalous pulmonary venous return). Age related effects were observed, with limited evidence of QTc prolongation in those under 25 years (7). Congenital heart defects were also reported in a fetus carrying a duplication of 4q25-ter and 9pter-q31.1 with breakpoints in chromosome four transecting *ANK2*; the fetus was born with multiple cardiac malformations including a large atrioventricular septal defect (39). However, it is unclear whether the congenital heart defects may be related to the duplications or whether *ANK2* haploinsufficiency played a role. Carriers of the balanced translocation, which includes breakpoints transecting *ANK2*, did not have congenital heart defects but other cardiac features including bradycardia, ventricular ectopy, sinus node dysfunction, and mild left ventricular dysfunction (Table 1).

LQTS is not the only phenotype associated with loss of ANKB function. Over time, several other arrhythmias and conduction anomalies have been associated with *ANK2* variants including catecholaminergic polymorphic ventricular tachycardia (CPVT), bradycardia, and WPW. An occurrence of CPVT in carriers of an *ANK2* variant has been reported in a small number of cases, with a recent report by Song et al. (40) of a 20 y.o. man with diagnosis of CPVT and non-ischemic cardiomyopathy who was found to carry the p.I3437T variant located in the disordered domain of ANKB (8, 41). WPW has been suggested to be another feature of Ankyrin-B syndrome. In addition to the one individual with WPW carrying the p.S646F variant previously mentioned, two rare de novo and one inherited variant in *ANK2* were identified in a cohort of patients with WPW (7, 42).

Beyond inherited arrhythmias, loss-of-function variants in *ANK2* have also been associated with cardiomyopathy, such as hypertrophic cardiomyopathy, dilated cardiomyopathy, and LV dysfunction (7, 39, 43). In a cohort of patients with HCM, rare variants in *ANK2* showed a statistically significant association with greater maximal mean left ventricular wall thickness, contributing to more severe LV hypertrophy (43). Recently, the role of *ANK2* in arrhythmogenic right ventricular cardiomyopathy (ARVC), a condition characterized by fibrofatty replacement of the myocardium, ventricular arrhythmias, and sudden cardiac death has come to attention. The previously reported p.E1458G variant was identified in an individual who died suddenly while running and was found to have ARVC on autopsy. A second *ANK2* variant was identified in a family where the proband died suddenly during exercise and was also found to have ARVC on autopsy. Post-mortem genetic testing was carried out and identified a novel p. M1988T variant, which is located in the C-terminal domain. Additional family members were identified through cascade screening to have definite or borderline diagnoses of ARVC (44).

With the emergence of a broad spectrum of features linked to *ANK2* variants came the term “Ankyrin-B syndrome,” which at the time more fully captured the complexity of the range of associated phenotypes (8, 32). Although the origin of Ankyrin-B syndrome is associated solely with cardiac phenotypes (8, 45), through the investigation and identification of new variants, it has become apparent that ANKB dysfunction is not exclusive to cardiac phenotypes but underlies neurological ones as well. Thus the term, Ankyrin-B syndrome, does not fully capture the broad spectrum of ANKB dysfunction across all cell types and variants. The pleiotropic nature of *ANK2* is highlighted by individuals that experience seizures in combination to the cardiac manifestations (7, 46), as well as unique *ANK2* variants associated with ASD (47–49) of which we will discuss in the next section.

### ANK2 variants associated with neurological phenotypes

Beyond the heart, *ANK2* is emerging as an important gene in neurological conditions, including ASD and epilepsy. It is important to note that the variants associated with Ankyrin-B syndrome (cardiac-phenotype associated ANKB variants) are distinct from those reported in association with ASD, and a combination of cardiac and ASD phenotypes has not been reported. Rare variants in *ANK2* including missense, frameshift, non-sense, and copy number variants have been identified in individuals with ASD (Table 2) (10, 47–50). *ANK2* is classified as a high-confidence gene clearly implicated in ASD by the Simons Foundation Autism Research initiative due to the reports of at least three de-*novo* loss-of-function variants in the literature and meeting the threshold false discovery rate of <0.1 (https://gene.sfari.org/database/human-gene/ANK2). ASD-associated *ANK2* variants are largely non-syndromic and typically not associated with intellectual disability (Table 2) (51). While some variants are present within both the 220-kDa and 440 kDa ANKB proteins, certain variants are unique to the 440-kDa giant ANKB isoform. For instance, a knock-in mouse model carrying ANKB p.P2580fs (analogous to the human p.R2608fs), which expresses a truncated giant ANKB polypeptide, demonstrated ASD-like behaviors including repetitive behavior, decreased ultrasonic vocalization, reduced territory marking, and superior executive functioning. Of note, mice homozygous and heterozygous for the p.P2580fs variant exhibited the same behaviors, supporting that haploinsufficiency of *ANK2* could contribute to risk for ASD (51). Using a multiplex network that characterized modules of epilepsy and ASD genes sharing similar phenotypes and protein-protein interactions, *ANK2* has also been identified as a novel candidate gene for epilepsy (11). Similarly, in a workflow using the random walk with restart algorithm in addition to permutation and functional association tests *ANK2* was also predicted as a novel gene for epilepsy (52).

**Table 2:** ANK2 variants associated with autism spectrum disorder.

**Variant**	**Type**	**Location**	**ASD**	**Intellectual disability**	**Other**	**gnomAD**	**Clinvar ID**	**Reference**
**Affect only giant ANKB** ***(440 kDa)*** **isoform**								
P1843S	Missense	Disordered	X			0.000003979		(134)
E3429V	Missense	Disordered	X			Absent		(48)
R2608fs	Frameshift	Disordered			Pervasive developmental disorder	Absent		(135)
**Affect giant ANKB** ***(440 kDa)*** **and** ***220 kDa*** **isoform**								
R990*	Nonsense	ZU5-1			Asperger's disorder	Absent	VCV000450028.2	(10)
Q1589Kfs	Frameshift		X	X	Sensorimotor neuropathy, facial dysmorphism	Absent	VCV000235896.1	(50)
4:113593803_113967887dup	Duplication		X			n/a	VCV000236353.1	(47)
4:114077690_118094709dup	Duplication		X			n/a	VCV000236354.1	(47)
4: 114225715-114429181del	Deletion		X			n/a	VCV000236355.1	(47)

Table adapted from Yang et al. (51); ASD, Autism Spectrum Disorder.

Notably, independent of the connection between *ANK2* variants and risk for epilepsy, seizures were reported in association with cardiac-phenotype associated *ANK2* variants. A history of seizures was reported in eight of eighteen carriers of the ANKB p.S646F variant, and in two out of six patients carrying the ANKB p.W1535R variant (7, 46). In a study which sequenced cases of epilepsy-related sudden unexpected death for inherited heart disease related genes, one individual was found to carry two variants in *ANK2* (p.Ser105Thr, p.Glu1934Val). Of note, this death occurred by drowning, and the individual was reported to have mildly prolonged QTc (53). Given that seizures can be linked to cardiac arrhythmias (54) and the fact that some cardiac-associated *ANK2* variants are linked with seizures (7, 46) it would be worth investigation to determine if the seizures are a result of the arrhythmia or independent and owed to dysfunction in the brain. Furthermore, with the link to epilepsy in neurological-associated *ANK2* variants (11) it raises the question of how ANKB dysfunction is impacting neuronal mechanisms.

### ANK2 variants associated with metabolic phenotypes

*ANK2* variants have also been implicated in the regulation of fat and glucose metabolism. In particular, the *ANK2* p.R1788W variant, which is associated to cardiac phenotypes (Table 1), was enriched in individuals of white and Hispanic descent diagnosed with type 2 diabetes in the American Diabetes Association GENNID cohort. Moreover, the *ANK2* p.L1622I variant, associated with a less severe cardiac phenotype, is the most frequent *ANK2* variant (7.5%) in African Americans who carry up to a 2-fold increased risk for type 2 diabetes (19, 55, 56). Whether other primarily cardiac or neuronal *ANK2* variants also result in global or local metabolic disturbances remains to be investigated.

Fundamental research studies revealing the many roles of ANKB within cells have provided insights into possible mechanisms behind the various phenotypes associated with *ANK2* variants. ANKB is implicated in different pathways, as it is a scaffolding protein for ion channels and transporters as well as a link for structural and signaling proteins, some of which are outlined below and summarized in Table 3.

**Table 3:** ANKB interacting partners.

**Domain**	**Classification**	**Interacting partner**	**Cell type interaction confirmed in**	**References**
MBD	Ion channels/ Transporters	Inositol 1,4,5-trisphosphate receptor	Cardiac and Neuronal	(4, 5, 14, 60, 103, 136)
		Ca_V_1.3	Cardiac	(136, 137)
		Ca_V_2.1	Neuronal	(101, 102)
		Ca_V_2.2	Neuronal	(101)
		Ca_V_3.1	Neuronal	(138)
		Ca_V_3.2	Neuronal	(138)
		Kir6.2	Cardiac	(139, 140)
		Sodium Calcium Exchanger	Cardiac and Neuronal	(4, 5, 24, 60, 103, 137)
		Sodium Potassium ATPase (a1 and a2)	Cardiac and Neuronal	(4, 5, 60, 103, 137, 140, 141)
		Erythrocyte anion channel	Neuronal	(12)
	Structural	EHD1-4	Cardiac	(142)
	Structural/ Signaling	Beta-catenin	Cardiac	(44)
	Cell adhesion	L1CAM	Neuronal	(51, 120, 143)
		Dystrophin	Cardiac	(144, 145)
SBD	Motor movement	Dynactin-4	Cardiac and Neuronal	(144–146)
	Structural	βII-spectrin	Cardiac and Neuronal	(6, 59, 103, 147)
	Signaling	Phosphatidylinositol 3-phosphate	Neuronal and Fibroblast	(146, 148)
		PP2A B56α	Cardiac	(95, 149, 150)
Giant insertion	Enzyme	Ndel1	Neuronal	(151)
DD	Signaling	RABGAP1L	Fibroblast	(148)
CTD	Chaperone	HSP40	Cardiac	(69)
	Signaling	Obscurin	Cardiac	(24, 152)
	Regulatory	Ankyrin-B MBD	Cardiac	(15)
Unknown	Signaling	SadA/SadB	Neuronal	(153)
	Chaperone	UNC-119	Neuronal	(80)

## Insights on the cellular role(s) of ANKB from model organism studies

**Figure 2:**
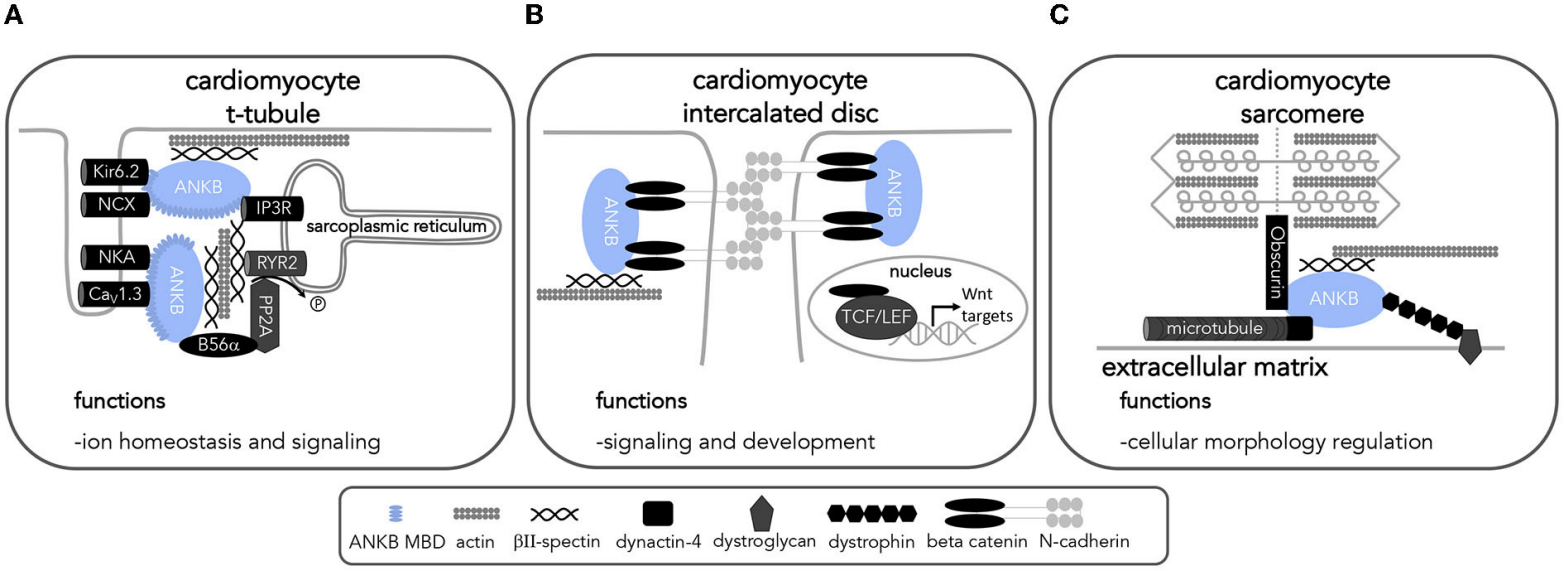
ANKB interactions in cardiomyocytes implicates ANKB in a variety of cellular processes. Diagram of ANKB interacting partners and their localization allowing for proper cardiac function. ANKB interactions at the **(A)** t-tubule **(B)** intercalated disc and **(C)** sarcomere allow for proper cell functions. Kir6.2, inward rectifier potassium channel; NCX, sodium calcium exchanger; NKA, sodium potassium ATPase; CaV1.3, voltage-gated calcium channel; IP3R, Inositol 1,4,5-trisphosphate receptor; PP2A, protein phosphatase 2A; RYR2, ryanodine receptor 2; L1CAM, L1 cell adhesion molecule; PI3P, phosphatidylinositol 3-phosphate; CaV1.3, voltage-gated calcium channel; TCF/LEF, T cell factor/lymphoid enhancer factor transcription factors.

Given their sequence similarity, it is possible to understand the biological role of *ANK2* (and its homologs) through studying model organisms. Mouse *Ank2* is comprised of exons exhibiting considerable homology to those found in human *ANK2* and exhibits similar tissue-specific isoform expression patterns (24, 25). In mice, global *Ank2* knockout causes neonatal death (57), while conditional *Ank2* knockout in the heart and brain results in significant electrical and structural impairments and death (44, 51, 58, 59). Heterozygous *Ank2* knockout (*Ank2*^+/−^) mice model haploinsufficiency (i.e., expression of a single wildtype *Ank2* allele fails to produce a wildtype phenotype), are relatively viable, and therefore used in many preclinical studies. *Ank2*^+/−^ mice display increased susceptibility to atrial and catecholamine-induced ventricular arrhythmias and sudden death, as well as, premature senescence and reduced lifespan (4, 8, 45). These cardiac manifestations have been associated with decreased presence of the sodium calcium exchanger, the sodium potassium ATPase subunits 1 and 2, and the inositol 1,4,5-trisphosphate receptor at the T-tubules of cultured primary cardiomyocytes (4, 60) (Figure 2). Mice with complete global loss of *Ank2* (*Ank2*^−/−^) display severe structural brain defects, such as hypoplasia of white matter tracts, dilated ventricles, and degeneration of the optic nerve (57). As several developmental signaling pathways are strongly intertwined with the homeostasis of ions, such as calcium (61–63), the severe structural phenotypes observed in the context of ANKB dysfunction (or haploinsufficiency) may be related to ANKB regulatory roles in ion homeostasis and cytoskeletal proteins.

Analysis of the molecular evolution of ankyrins has revealed a single *ankyrin* gene in *Caenorhabditis elegans* (*unc-44*), two *ankyrin* genes in *Drosophila melanogaster* (*Dank1, Dank2*), and three mammalian *ankyrin* genes (*ANK1, ANK2, ANK3*) that likely originated from a single *ankyrin* ancestor gene in *Ciona intestinalis* (64, 65). Moreover, these analyses have also demonstrated a closer evolutionary relationship between *ANK2* and *ANK3*, which despite their high sequence and structural similarity localize to different cellular compartments and associate with different proteins (66, 67). In most cases, ankyrins do not have the ability to compensate for each other (68, 69). However, previous studies from the Rasband lab have shown that in the central nervous system ANKB can partially compensate for loss of paranodal ANKG and ANKR can compensate ANKG's role in sodium channel clustering at nodes of Ranvier (70, 71). While each ankyrin protein appears to have different roles they share some protein-protein interactions and can provide insight into each other's roles in cells.

**Figure 3:**
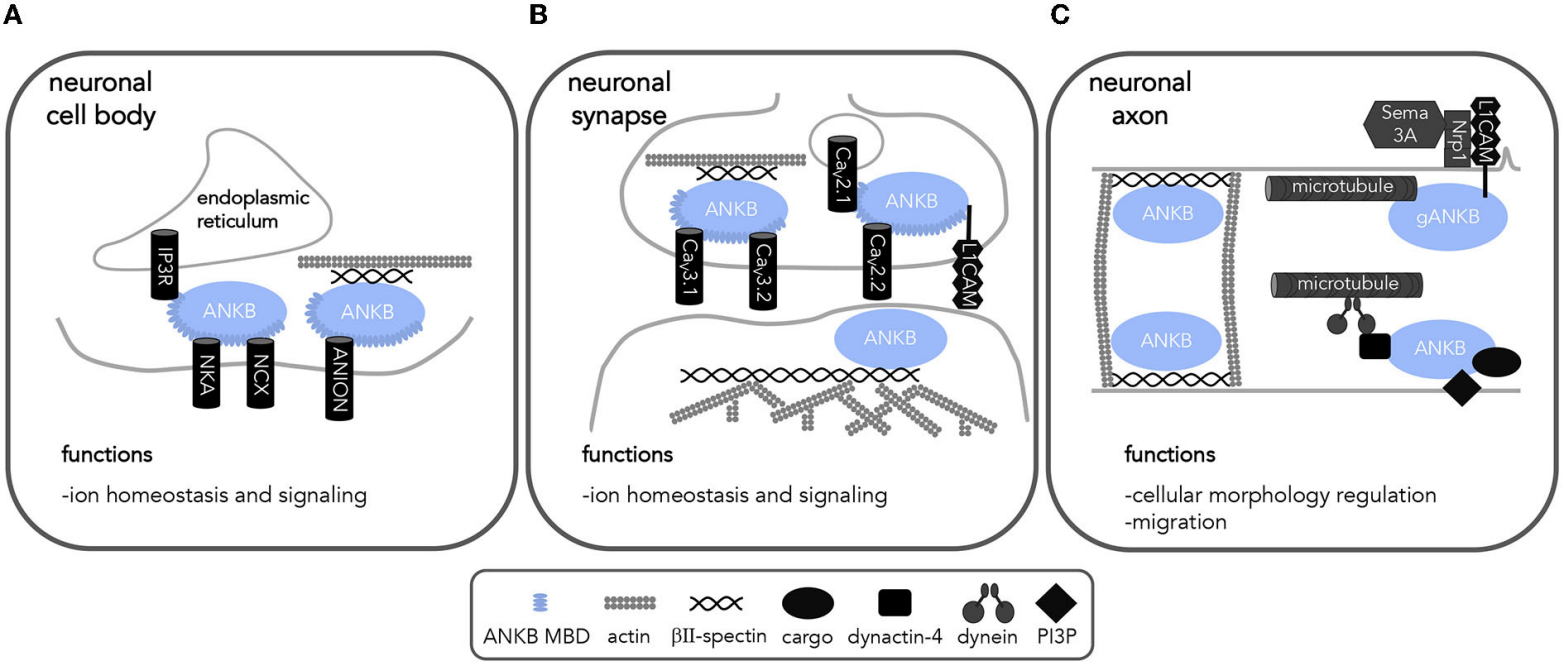
ANKB interactions in neurons implicates ANKB in a variety of cellular processes. Diagram of ANKB interacting partners and their localization allowing for proper neuronal function. **(A)** cell body **(B)** synapse and **(C)** axon allow for proper cell functions. NCX, sodium calcium exchanger; NKA, sodium potassium ATPase; IP3R, Inositol 1,4,5-trisphosphate receptor; Ca_V_, voltage-gated calcium channels; PP2A, protein phosphatase 2A; RYR2, ryanodine receptor 2; L1CAM, L1 cell adhesion molecule; PI3P, phosphatidylinositol 3-phospate; Sema 3A, semaphorin 3A; Nrp1, semaphorin 3A receptor neuropilin 1.

The use of model organisms can help unravel the mechanisms behind the clinical phenotypes associated with *ANK2* variants. Insights from model organisms have elucidated ANKB's essential roles in regulating cellular morphology, polarization, calcium homeostasis, and glucose and fat metabolism, as outlined below and summarized in Tables 3–**5** and Figures 2, 3.

**Table 4:** Summary of ANKB's cellular roles identified using model organisms.

**Model organism**		**Biological process**	**Elucidated roles and implications**	**Reference**
*Mus*	*Ank*2^−/−^ (*Ank2* null)	N/a	Global knockout is deadly	(57)
*musculus*	*Ank*2^+/−^ (Models haploinsufficiency)	Cardiomyocyte structural development	Cardiac malformations imply role in structural development	(4, 8, 45)
		Calcium homeostasis and signaling	Localization and expression of the sodium/calcium exchanger, inositol trisphosphate receptor, and voltage-gated calcium channels L-type channels; Ca_v_1.3 expression (SAN isolated cells and atrial cardiomyocytes) P/Q-type channels; Ca_v_2.1 and Ca_v_2.2 expression (cortex, cerebellum, and brainstem)	(4, 60, 101, 136, 137)
			Regulation of RYR2-mediated sarcoplasmic reticulum calcium leak *via* PP2A (cardiomyocytes)	(104)
			Regulation of calcium homeostasis affects calcium cycling dynamics (calcium transients, sparks) and delayed afterdepolarizations	(4, 5, 95, 104)
		Glucose and fat metabolism	Downstream effects on oral glucose tolerance	(114)
	shAnkB knockdown	Calcium homeostasis and signaling	Localization and expression of T-type channels Ca_v_3.2 expression (hippocampal neurons)	(138)
	Cardiac-specific conditional knockout	Cardiomyocyte structural development	Cardiac remodeling implies structural role	(44)
			Involved in Beta-catenin localization and expression; possible implications on beta-catenin/Wnt signaling	
	Brain-specific knockout (brain-specific ANKB 440-kDa isoform not expressed)	Neuronal structural development	Synaptic signaling and synapse excitability	(51)
			Axon branching and connectivity (linked to *Ank2* involvement in microtubule bundle formation)	
			Abnormal social behavior. Impaired communicative behavior. Enhanced executive function.	
	Excitatory neuron-specific knockout (ANKB 220-kDa and 440-kDa are not expressed in excitatory neurons)	Calcium homeostasis and signaling	Regulation of Cav2.1 expression (decreased Cav2.1 expression in whole cortex homogenates)	(102)
	Adipose tissue-specific conditional knockout	Glucose and fat metabolism	Adiposity	(117)
			Pancreatic islet size	
			Insulin resistance	
	*ANK2* p.R1788W knock-in	Glucose and fat metabolism	Abnormal insulin secretion. Insulin resistance	(19)
			Increased peripheral glucose uptake (increased cell surface GLUT4)	
			Adiposity	
*Caenorhabditis elegans: unc-44*		Neuronal development and polarization	Regulating organization/ polarization neurite microtubule networks	(79, 81)
*Drosophila melanogaster: Dank2 (Dmel\ank2)*		Neuronal development and polarization	Supporting stabilization and remodeling of synaptic microtubule network	(87, 88)

**Table 5:** Summary of primary biological functions affected by ANKB dysfunction/loss-of-function.

**Biological function**	**Level**	**Heart**	**Brain**
Structural development and cell polarization	Cellular	Trafficking and distribution of ion channels and exchangers along T-tubules and beta-catenin at the intercalated disc	Definition of axonal and dendritic compartments in neurons Trafficking of proteins to axonal and dendritic compartments
	Tissue/Organ	Dilated cardiomyopathy Ventricular wall fibrosis	White matter tract defects Increased axonal connectivity
Calcium homeostasis and signaling	Cellular	Increased calcium transient amplitude (putatively, increased intracellular calcium concentration) Increased calcium sparks (calcium release events from the sarcoplasmic reticulum) Decreased calcium transient frequency Decreased spontaneous contraction rate	Increased miniature excitatory postsynaptic potentials Decreased excitability Decreased action potential firing rate
	Tissue/Organ	Increased contractility Increased rate of delayed afterdepolarizations	Decreased expression of calcium voltage gated channels (Ca_V_2.1 and Ca_V_2.2)

### ANKB regulates cellular morphology and polarization

As cells develop, migrate, and mature, cytoskeletal rearrangements lead to the specification of a directionality axis resulting in well-organized regions that support motility, cell-to-cell contacts, and surfaces for secretion or absorption (72). This spatiotemporal phenomenon is known as cell polarity and it is what influences the shape, motility, and trafficking and signaling domains in cells, as well as their ability to respond and adapt to extracellular and intracellular signals (72, 73). In the mouse heart, for example, cell polarization allows round embryonic ventricular cardiomyocytes to postnatally adopt the shape of a rod and direct their junctional proteins to the ends of the now elongated cells to form the intercalated disc, a specialization for cell-to-cell communication (74, 75). In other cell types, such as neurons, cell polarization defines specialized compartments for receiving (dendrites) and sending (axons) electrochemical signals (76). Insights from studies using model organisms have shown ANKB's essential role in neuronal development which raises the question of how ANKB may play a role in cardiomyocyte development as well.

Although it is yet unclear whether ANKB plays a role in the morphological development of cardiomyocytes and other cardiac cell-types in which it is expressed, there is evidence of ANKB's roles in neuronal development. Studies using the model organism *C. elegans* have demonstrated that *ANK2*'s ortholog *unc-44*/ankyrin is a known master regulator cell polarization and axonal neurite outgrowth in this roundworm's sensory neurons (77–80). Mutations affecting *unc-44*/ankyrin function result in abnormal neural development, locomotor defects, and microtubule networks with mixed polarity in axons and dendrites leading to abnormal protein sorting and trafficking into these compartments (77–79, 81–83). *Unc-44*/ankyrin, along with *unc-33*/crmp (an actin and microtubule associating protein) and *unc-116*/kinesin-1 (a motor protein) help establish neuronal polarity by regulating the organization of dendritic and axonal microtubule networks (79, 81). Furthermore, *unc-44*/ankyrin acts upstream of *unc-33*/crmp and *vab-8*/kinesin-like protein to regulate the removal of gap junction channels (84) which allow for the direct electrical communication between cells and play a key role in development (85, 86). In *Drosophila melanogaster, Dmel*\*ank2*, which has a short and long/ giant isoform localized to different sub-cellular compartments (cell body and axon, respectively) supports the stabilization and remodeling of the synaptic microtubule network (87, 88). Loss of *Dmel*\*ank2* results in retraction of synaptic boutons, collapse of the pre-synaptic active zones, reduction of the terminal size, and altered neuromuscular junction morphology (88, 89). While the role of *ANK2* in cardiomyocyte polarization during heart development remains to be investigated, some of the *ANK2* variants listed above have been associated with cardiac malformations suggesting that ANKB dysfunction results in an impact to the structural development of these cells (7, 39).

Recent organ-specific *ANK2* conditional knockouts further underscore the important role of *ANK2* in the structural development of cardiomyocytes and neurons. Specifically, the beta-catenin/Wnt signaling pathway is important in both cardiac and neuronal cell fate determination, axis patterning and polarity, and proliferation (90–92). This pathway is initiated by the accumulation of beta-catenin in the nucleus leading to the transcription of Wnt responsive genes (92). Evidence underlying ANKB's role in cell proliferation and survival has been highlighted by the p.S646F variant. In H9c2 cells, a cell model with similar traits of primary cardiomyocytes (93), expression of the p.S646F variant resulted in decreased cellular viability and proliferation (94). Using a cardiac-specific conditional knockout model, Roberts *et al*. found that loss of *Ank2* in the heart leads to severe cardiac remodeling resulting in ventricular dilation, fibrosis, bradycardia, QTc prolongation, and increased susceptibility to catecholamine-induced ventricular arrythmias (44). Associated with decreased protein expression and altered localization of beta-catenin away from the intercalated disk, this cardiac-specific *ANK2* knockout phenotype recapitulates what has been observed in arrhythmogenic cardiomyopathy phenotypes as well as in patients carrying predicted loss-of-function *ANK2* variants and their respective knock-in mouse models (Figure 2) (4, 37, 95). It is worth noting that cardiomyocytes with *Ank2* loss do not show altered expression nor mislocalization of intercalated disc proteins such as plakoglobin, plakophilin 2, connexin 43, N-cadherin, desmoplakin, and desmoglein 2 (44). Further insights regarding the involvement of ankyrin proteins in this context may be drawn from ANKG, which also interacts with beta-catenin. Loss of ANKG results in a comparable decrease in beta-catenin localization at the membrane and increased nuclear levels leading to an increase in neural progenitor proliferation in mice *via* Wnt signaling (96). Given ANKB also plays a role in organizing beta-catenin localization and expression, it is worth future investigations to determine if ANKB leads to any effects on Wnt signaling. In parallel, another ANKB interacting partner, protein phosphatase 2A (PP2A), is also a regulator of Wnt signaling (97). With ANKB's potential involvement at two stages of the Wnt signaling pathway, future studies should explore the implications of ANKB dysfunction on the latter as well as concomitant developmental processes.

In the case of loss of brain-specific giant ANKB 440-kDa, which primarily localizes to axons, mice display ectopic axon branching and connectivity, transient increase in excitatory synapses, and neurodevelopmental disorder-like behaviors such as stereotype movements and impaired social behavior (51). The impairment on axonal connectivity has been linked to *ANK2's* role in regulating the formation of microtubule bundles in the axon and reducing branching points enriched with F-actin by promoting growth cone collapse in response to semaphorin 3A signaling (58, 98) (Figure 3). While brain specific *Ank2* knockout mice do not exhibit impairments in memory and learning (51), the identified structural and connectivity changes recapitulate some of the morphological features observed in neurodevelopmental disorders, such as ASD (51, 99, 100). The identification of giant ANKB-specific roles in critical aspects of neuronal structural development warrants further exploration in the heart and its intrinsic nervous system. Furthermore, given that ANKB is associated with several critical steps in the development of cells and the establishment of their polarity, studies aiming to elucidate the role that *ANK2* plays during early heart and cardiac conduction system development will be crucial to understand the various phenotypes associated with *ANK2* variants.

### ANKB regulates calcium homeostasis

With its role in proper localization of the calcium the sodium/calcium exchanger, the inositol trisphosphate receptor, and calcium voltage-gated channels, ANKB is a key hub for regulation of calcium homeostasis in excitable cells (14, 101–103). In mouse cardiomyocytes, complete and partial loss of *Ank2* leads to abnormal calcium dynamics as summarized in Tables 4, 5 (4, 5, 104). Using global and partial loss of *Ank2* knockout mouse models, it has been demonstrated that *ANK2* variants identified in cardiogenetic studies have differential effects on cardiomyocyte calcium dynamics *in vitro*, with some variants (namely: p.G1406C, p.R1450W, p.L1503V) rescuing calcium transient amplitude defects, while others (namely: p.E1425G, p.L1622I, p.T1626N, p.R1788W, p.T1404I, p.V1516D, p.T1552N, p.V1777M, and p.E1813K) fail to rescue calcium and spontaneous activity abnormalities (8). These *in vitro* experimental findings are in line the variable expressivity and penetrance observed in individuals carrying *ANK2* variants (4, 7).

*ANK2* regulates calcium homeostasis in excitable cells through various potential mechanisms, some of which still require additional in-depth characterization. Loss of *ANK2* or *ANK2* dysfunction (as in the case of pathogenic *ANK2* variants) leads to the mis-localization of channels and transporters involved in calcium handling (sodium/calcium exchanger, inositol trisphosphate receptor, and calcium voltage-gated channels) (4, 7, 95, 105). Furthermore, lack of *ANK2* (and in some cases, *ANK2* dysfunction) leads to decreased protein expression of the sodium/calcium exchanger and L-type, T-type, and P/Q-type voltage gated calcium channels in cardiomyocytes and neurons (7, 101, 102, 104). Specifically, two clinically relevant ANKB variants, p.E1458G and p.S646F, differentially modulate levels of Ca_V_2.1, the pore forming subunit of P/Q-type voltage gated calcium channels, in HEK293T cells (102). The p.E1458G variant was found to decrease surface Ca_V_2.1 levels while the p.S646F variant increased intracellular Ca_V_2.1 levels. Another variant, p.Q879R, which to our knowledge has not yet been associated with disease, is located at the linker region required for proper ANKB localization. Expression of p.Q879R increased the surface level expression of Ca_V_2.1 in the presence of the Ca_V_ accessory subunits (102). Additionally, ANKB may also regulate the key intracellular calcium release channel, ryanodine receptor 2 (RYR2). RYR2 hyperphosphorylation in the mouse *Ank2* knock-in model harboring the p.Q1283H variant suggests ANKB's interaction with the regulatory subunit, B56α, of the protein phosphatase PP2A, of the protein phosphatase PP2A (*PPP2R5A*) is necessary for PP2A activity on RYR2 (Figure 2) (95). Abnormal calcium handling associated with ANKB variant expression is a plausible pathophysiological mechanism underlying the increase in frequency of delayed afterdepolarizations and susceptibility for cardiac arrhythmias observed with *ANK2* cardiac variants (104, 106), as well as a possible mechanism for the increased susceptibility to epileptic seizures associated with some *ANK2* variants.

PP2A is a key regulator in most signal transduction pathways and cellular processes (107, 108). Other targets of PP2A and the resulting impact of ANKB dysfunction has not yet been investigated and should be an area of research in the future. Of the many PP2A targets some include other ANKB interactors such as the inositol trisphosphate receptor (109) and the sodium potassium ATPase (110) of whose phosphorylation and therefore function may also be altered as a result of ANKB dysfunction. In neuronal cells PP2A is one of the major enzymes associated with regulating microtubules, neurofilaments, and the actin cytoskeleton (111–113). While ANKB's interaction with PP2A in neurons has not yet been confirmed, this likely regulation of signaling events has key implications to the functioning and development of neuronal cells as well.

### ANKB functions in glucose and fat metabolism regulation

ANKB has also been linked to regulating glucose and fat metabolism. An earlier study by Healy et al. (114) described that mice with partial global loss of *Ank2* (*Ank2*^+/−^) exhibit impaired oral glucose tolerance likely secondary to decreased expression of the inositol trisphosphate receptor in pancreatic islets, which mediates the signaling for augmented glucose-induced insulin secretion after parasympathetic stimulation (115, 116). Knock-in mice harboring the type-2 diabetes-associated *ANK2* p.R1788W variant exhibit decreased insulin secretion following parasympathetic stimulation and increased peripheral glucose uptake (coupled with increased plasma membrane density of the glucose co-transporter 4 in skeletal muscle and adipose tissue) (19). Notably, older *ANK2* R1788W mice had increased adiposity and showed insulin resistance (19). The increase in adiposity is also observed in adipose tissue-specific *Ank2* knockout mice, which develop progressive pancreatic islet dysfunction, accumulation of fat with age or high fat diet, and insulin resistance associated with impaired glucose co-transporter clathrin-mediated endocytosis (117). Importantly, a subset of *ANK2* variants associated with cardiac arrhythmias failed to rescue the metabolic defects in *Ank2*^−/−^ adipocytes (117), calling to attention additional cardiovascular risk considerations for individuals with known *ANK2* cardiac arrhythmia variants. A knock in *ANK2* p.L1622I model exhibited a measurable and distinct cardiac phenotype, reduced ANKB expression, and even developed insulin resistance and age-dependent increases in adiposity (19).

## Discussion

Variants in *ANK2* are associated primarily to complex cardiac phenotypes; however, some functional *ANK2* variants also have neurological or metabolic manifestations. Cardiac phenotypes associated with *ANK2* functional variants are characterized by a predisposition to arrhythmias, conduction anomalies, and congenital and adult-onset structural heart disease, and in some cases, seizure (Table 1). Other *ANK2* variants may contribute to risk for ASD and epilepsy (Table 2). With type 2 diabetes also linked to specific *ANK2* antecedents, the putative compounding effects of metabolic perturbation on cardiac and neurological phenotypes may pose additional risk to individuals carrying *ANK2* variants. The diversity of manifestations associated with *ANK2* variants could result, in part, from complex ANKB protein interaction networks involving critical proteins that regulate cellular structure and function (Table 3). Overall, improved knowledge of ANKB cellular roles and regulation is now needed to advance understanding of clinical phenotypes associated with *ANK2* variants and, ultimately to develop improved, targeted therapeutic approaches.

As there is such diversity in features reported across *ANK2* variants with cardiac phenotypes, further studies are required to better understand which features are truly linked to Ankyrin-B syndrome. For example, congenital heart defects have been described in association with only one variant to date, p.S464F (7), and a structural chromosomal re-arrangement involving breakpoints in *ANK2* (39). Whether congenital heart defects are part of the ANKB spectrum of manifestations or just isolated events remains to be determined. In favor of the notion that *ANK2* functional variants can also contribute to structural heart disease, a British study on hypertrophic cardiomyopathy reported that the proportion of patients with a maximum left ventricular wall thickness >30 mm (i.e., extreme wall thickness) was higher in carriers of *ANK2* variants (43). This effect was still present when restraining the analysis to patients carrying sarcomeric protein variants (43), suggesting that *ANK2* might play a role of a disease modifier in cases of hypertrophic cardiomyopathy (43, 118, 119). Further population and laboratory studies are required to fully elucidate the connection between *ANK2* variants and hypertrophic cardiomyopathy, which could involve ANKB interactions with structural/cytoskeletal elements within cardiac cells.

Given the cases of structural malformations it is also important to investigate the potential role of ANKB in cardiac cell development. Of note, the p.S6464F variant is less stable and experiences reduced expression only in undifferentiated H9c2 cells suggesting this variant's impact to cells occurs during their development (94) and provides some additional rationale behind investigating ANKB's roles during cardiac development. As seen in patients with both the p.S646F (7) and p.E1458G (4) variants there appears to be an age-related effect. This implies that not only is ANKB function important in early development but also over a lifespan. Some possible mechanisms behind ANKB's role in cardiac development include its interactions with beta-catenin, PP2A, and ion channels. Understanding the developmental expression of ANKB and the impact of variants may provide insight into the cardiac dysfunction observed in patients over their lifetime.

While ANKB's link to neuronal development has been better pieced together through studies with model organisms, we have highlighted key knowledge gaps and areas of future investigations. Early observations revealed neuroanatomical defects in the global *Ank2* knockout mouse (57) and model organisms have highlighted homologous ankyrin roles in neuronal polarization (79). Other mainly *in vitro* studies point to a role for ANKB in GABAergic synaptic development (120), axonal branching (51, 58, 98), and voltage-gated calcium channel trafficking (101, 102) (Figure 3). These studies suggest ANKB regulates neurodevelopmental processes and could help explain its putative role in risk for ASD, as well as its association with epilepsy and seizure. Given the important roles of giant ANKB in the development of the nervous system, future studies aiming to elucidate the roles of giant ANKB in the development of the heart and conduction system are warranted. Moreover, recent single cell transcriptomics surveys identifying several non-myocyte cells that contribute to heart development, such as cardiac neural crest cells, neuronal cells, and glial-like cells (all with detectable *ANK2* transcripts levels) (26, 121–123) open the door to novel lines of research investigating *ANK2*'s functions within these cells and their impact in shaping heart development. Notably, many studies in the brain have focused primarily on the giant isoform of ANKB, the putative central nervous system-specific, neonatal isoform (51, 58, 98); however, the roles of the smaller, more prominent 220 kDa isoform are vastly understudied. ANKB's roles at the mammalian synapse are yet to be studied even though ANKB is not only enriched at synapses, but also seems to associate with multiple postsynaptic scaffolding proteins (124, 125). Given ANKB's interactions with ion channels, βII-spectrin, and components of the cytoskeleton (126), it is possible that ANKB plays important roles in regulating the shape of postsynaptic structures and protein sorting therein contributing to maturation of synapses and establishment of neuronal circuits.

Recently, disease associations of *ANK2* variants and LQTS and CPVT have been debated in part due to the population frequency of certain previously reported variants (34–36, 127). Although the minor allele frequency is certainly a useful predictive tool (128), an elevated minor allele frequency may not completely eliminate a role for the variants in disease. For example, while the *ANK2* p.L1622I variant is associated with prolonged QTc and ventricular tachyarrhythmias, which is modeled in a knock-in homozygous mouse, the study was limited by the use of juvenile homozygous mice. This cardiac phenotype likely exceeds that of the carriers in the general population, who are most likely heterozygous for the *ANK2* p.L1622I variant (37). It is possible that *ANK2* variants are part of an oligogenic/polygenic disease (129). Such a possibility is seen with the p.E1813K variant which has been shown to aggravate the cardiac phenotype of an individual carrying KCNH2 p.H562R variant (130). In isolation, the p.E1813K variant was associated with age-related conduction disease, and the individual carrying only the KCNH2 p.H562R variant was asymptomatic. *ANK2* is a gene that appears to tolerate mutations well as seen by the allele frequencies of many variants. This variant toleration may be a result of a compensatory mechanism to protect the overall function of the protein given its apparent importance in cellular biology. Overall, this evidence highlights the importance of integrating allele frequency, genetic ancestry, and environmental and genetic factors in the analysis and determination of cardiovascular gene-disease associations of *ANK2* variants.

Insights from model organism studies have highlighted the significance of ANKB's many roles within cells. *ANK2* variants are linked with cardiac, neurological, or metabolomic phenotypes consisting of electrical, structural, and signaling impacts. The mechanisms behind *ANK2* variant dysfunction can be explained in part due to ANKB's protein interactions and cellular partners outlined within the review. With many interactions in both signaling and cytoskeletal components, ANKB can easily be implicated in a variety of cellular events and basic functions. Furthermore, interactions identified and studied within one cell type could hold relevance across multiple cell types in which ANKB is expressed. With the large number of ANKB protein-protein interactions the phenotype associated with one particular variant could be anticipated to be vastly different from another depending on the amino acid location and the degree of conservation (chemical similarity). A variant located within the membrane binding domain is likely to have a different phenotype than a variant located within the spectrin binding domain as an ion channel disruption will result in altered signaling compared to losing a structural interaction.

Improved understanding of ANKB cellular roles and the effects of variant expression at a mechanistic level is needed to advance the identification possible therapeutic targets and biomarkers for individuals with *ANK2* variants. Comprehensive characterization of ANKB's interacting and signaling partners would facilitate the design of small molecule modulators or repurposing of compounds to mitigate cellular pathology associated with ANKB variants. For example, inhibition of CamKII with KN-93 was able to mitigate RYR2 hyperphosphorylation and subsequent excessive calcium release in *Ank*2^+/−^ pro-arrhythmogenic mouse hearts, resulting in a net reduction of RYR2 phosphorylation, calcium spark frequency, and delayed afterdepolarizations (95, 131, 132). More recently, inhibition of the GSK-3β pathway with SB-216763 (resulting in a net activation of the Wnt/beta-catenin signaling cascade) was effective in ameliorating cardiac remodeling in mice presenting with arrhythmogenic cardiomyopathy associated with cardiac specific loss of ANKB (44). However, given ANKB expression in other excitable tissues and the important roles linked to signaling pathways in which ANKB directly or indirectly participates, it is paramount to continue advancing the understanding of ANKB's role in cells and molecular pathways before defining and launching ANKB-targeting therapeutic programs. This is particularly important given the limited mechanistic appreciation of neurological phenotypes associated with *ANK2* variants, such as seizure and white matter abnormalities. By exploiting the relatively conserved amino acid sequence and biological functions of ANKB and the availability of experimental model organisms, high-throughput cellular and molecular characterization of variants can bridge the gap to improved clinical understanding and development of targeted, specific therapeutic interventions.

The variability of clinical phenotypes associated with *ANK2* variants poses challenges for treatment. At present, the understanding of the source of this variability is incomplete but could be partly due to the pleiotropic effects of ANKB, as well as surreptitious layering of variants in related pathways and/or environmental factors. The complexity and incomplete mechanistic understanding of ANKB cellular roles and regulation pose significant challenges for development of precise therapeutic interventions. As technological advances in personalized and precision medicine continue to expand, successful therapeutic strategies will arise from testing and modeling *ANK2* variants directly on induced pluripotent stem cells derived from affected individuals themselves (133). A combination of experimental approaches, including personalized and precision medicine methods such as *in vitro* studies using patient-derived induced pluripotent stem cells, and model organism approaches will help to bridge the gaps to the identification of key pathways and therapeutics that target them safely and effectively. Current clinical efforts should therefore focus on monitoring carriers of *ANK2* functional variants for arrhythmia and cardiomyopathy, along with symptomatic and treatment and control of co-morbidities (106).

Highlighted within this review are a variety of *ANK2* variants and the different disease-linked phenotypes that arise as a result of their expression. Bringing together studies from model organisms and laboratory findings this review identifies potential mechanisms underlying ANKB dysfunction and possible contributions to disease. Investigating mechanisms underlying this link to disease will not only aid in our understanding of cellular pathways and ANKB's roles within them but will provide insight into disease risk and presentation. Understanding ANKB's roles in health and disease will advance the ability to translate this information into clinic and provide insights into developing treatments and therapies.

## Author contributions

Conceived by NY, LAS, and LTA. All authors wrote and revised the manuscript, contributed to the article, and approved the submitted version.

## Funding

This work was supported by a Canadian Institutes for Health Research Project Grant (CIHR; PJT-169064) awarded to LTA and LAS. NY was supported by a CIHR Graduate Scholarship-Master's Program scholarship and University of Victoria Donor Awards. JCSA was supported by a Michael Smith Health Research BC Trainee Award (RT-2021-1735). LTA was supported by a BC Children's Hospital Research Institute Investigator award.

## Conflict of interest

The authors declare that the research was conducted in the absence of any commercial or financial relationships that could be construed as a potential conflict of interest.

## Publisher's note

All claims expressed in this article are solely those of the authors and do not necessarily represent those of their affiliated organizations, or those of the publisher, the editors and the reviewers. Any product that may be evaluated in this article, or claim that may be made by its manufacturer, is not guaranteed or endorsed by the publisher.
